# Supplementary material for: Deep Multi-Modal Skin-Imaging-Based Information-Switching Network for Skin Lesion Recognition
Source: Bioengineering (Basel). 2025 Mar 12;12(3):282. doi: 10.3390/bioengineering12030282 (PMC11939189; doi:10.3390/bioengineering12030282)
Supplement: Supplementary file 1 [file bioengineering-12-00282-s001.zip › bioengineering-3423483-supplementary.pdf]

Supplementary Information

Deep Multi-Modal Skin-Imaging-Based Information-Switching Network for Skin Lesion Recognition

Yingzhe Yu [1,†], Huiqiong Jia [2,3,†], Li Zhang [4], Suling Xu [1], Xiaoxia Zhu [1], Jiucun Wang [5], Fangfang Wang [1], Lianyi Han [6], Haoqiang Jiang [6], Qiongyan Zhou [1,*] and Chao Xin [1,5,*]

1 The First Affiliated Hospital of Ningbo University, Ningbo 315211, China; yuyingzhe90@foxmail.com (Y.Y.); xusuling@nbu.edu.cn (S.X.); nb_zhuxiaoxia@126.com (X.Z.); fyywangfangfang@nbu.edu.cn (F.W.)
2 Department of Laboratory Medicine, The First Affiliated Hospital, Zhejiang University School of Medicine, Hangzhou 310000, China; jiahq@zju.edu.cn
3 Key Laboratory of Clinical In Vitro Diagnostic Techniques of Zhejiang Province, Hangzhou 310000, China
4 Department of Dermatology, The First Hospital of China Medical University, Shenyang 110001, China; lizhang_1001@126.com
5 State Key Laboratory of Genetic Engineering, Collaborative Innovation Center for Genetics and
Development, School of Life Sciences and Human Phenome Institute, Fudan University, Shanghai 200433, China; jcwang@fudan.edu.cn
6 Greater Bay Area Institute of Precision Medicine (Guangzhou), School of Life Sciences, Fudan University, Shanghai 315211, China; hanlianyi@fudan.edu.cn (L.H.); hqjiang23@m.fudan.edu.cn (H.J.)
* Correspondence: zhou.qiongyan@163.com (Q.Z.); xinchao_tech@163.com (C.X.)
† These authors contributed equally to this work.

Ablation study

Extensive ablation experiments have been carried out to evaluate the impact of different components on the performance of the suggested MDSIS-Net model. These tests make use of multi-modal DD and MM datasets, which allow for a comprehensive analysis of the behavior and responses of the model in different scenarios.

Influence of different modal fusion methods

In addition, this article compares the performance of our proposed MDSIS-Net model with different modal fusion methods. Specifically, we compare our multi-modal information-switching approach with early fusion and late fusion techniques. Early fusion involves converting the original three-channel RGB image input into an N×3 channel multi-modal input, where N represents the number of input modes for the DD and MM datasets. In the first convolutional layer, we modify the input channels from 3 to N×3, enabling early fusion. On the other hand, late fusion involves concatenating features at the final few layers of the neural network. Table S1 demonstrates the performance of the three methods using five metrics. On the DD dataset, our approach achieves the best performance with an mAP increase of 1.4% and 1.7% compared to the other two methods, an accuracy increase of 6.0% and 8.3%, a precision increase of 6.0% and 10.2%, a recall increase of 3.0% and 4.3%, and a f1-score increase of 4.6% and 7.4%. On the MM dataset, we observe an mAP increase of 6.8% and 4.0%, an accuracy increase of 5.9% and 3.3%, a precision increase of 12.4% and 8.1%, a recall increase of 5.9% and 2.1%, and a f1-score increase of 8.2% and 4.2%. These results indicate that the information exchange-based approach outperforms the other two methods.

Table S1. Ablation study on the different feature fusion methods for DD and MM datasets.

Fusion method	mAP	Accuracy	Precision	Recall	F1-score	Dataset
Early fusion framework	0.953	0.900	0.875	0.930	0.901	DD
Late fusion framework	0.950	0.877	0.833	0.917	0.873	DD
Information switching framework	**0.967**	**0.960**	**0.935**	**0.960**	**0.947**	DD
Early fusion framework	0.809	0.848	0.787	0.756	0.769	MM
Late fusion framework	0.837	0.874	0.830	0.794	0.809	MM
Information switching framework	**0.877**	**0.907**	**0.911**	**0.815**	**0.851**	MM

Figure S1 displays the visualization results of Grad-CAM under different fusion methods on the DD dataset. The early fusion visualizations exhibit identical results for each modality, which lacks interpretability in clinical practice since the disease focus points vary across different imaging patterns for DD. In contrast, the late fusion visualizations do not reveal the presence of inflammation in the red marks. However, our proposed information-switching model accurately captures inflammatory regions, as indicated by the red regions in Figure S1, and also focuses on subtle spots within UV spots and porphyrins. Therefore, the information-switching model demonstrates superior interpretability on DD compared to the other two methods.

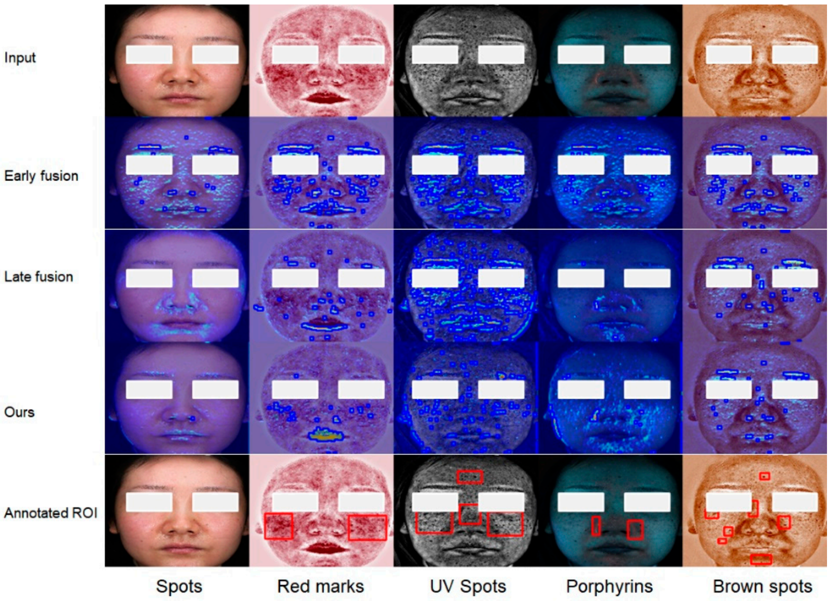

Figure S1. Comparison of the feature maps on different fusion methods for the DD dataset.

Figure S2 illustrates the visualization outcomes of Grad-CAM using various fusion techniques on the MM dataset. Both early and late fusion methods exhibit limited interpretability in clinical applications. In contrast, our proposed information-switching model effectively incorporates information from both clinical and dermoscopic images, accurately highlighting regular pigmented regions and asymmetric rash areas, as depicted by the red regions in Figure S2. Consequently, the information-switching model showcases superior interpretability for MM compared to the other fusion methods.

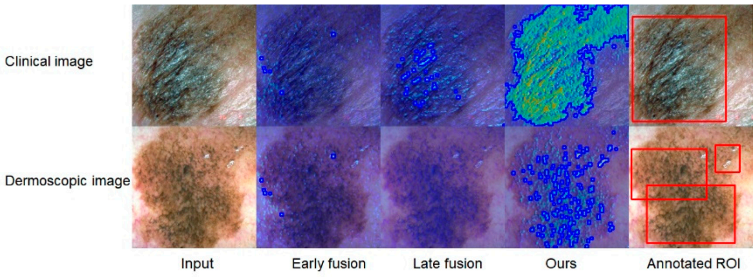

Figure S2. Comparison of the feature maps on different fusion methods for the MM dataset.

Figure S3 showcases the t-SNE visualization results of the DD dataset under different fusion methods. In the early fusion model's classification results, some features of melasma samples are mixed into the acne cluster. In the fusion early model's classification results, there are more misidentifications between the dermatitis and melasma clusters. On the other hand, the information-switching model not only separates the acne cluster from the other two clusters but also exhibits fewer misidentifications between the dermatitis and melasma clusters. This result indicates the advantage of the information-switching model in improving cluster separation and reducing misclassifications on DD between different clusters.

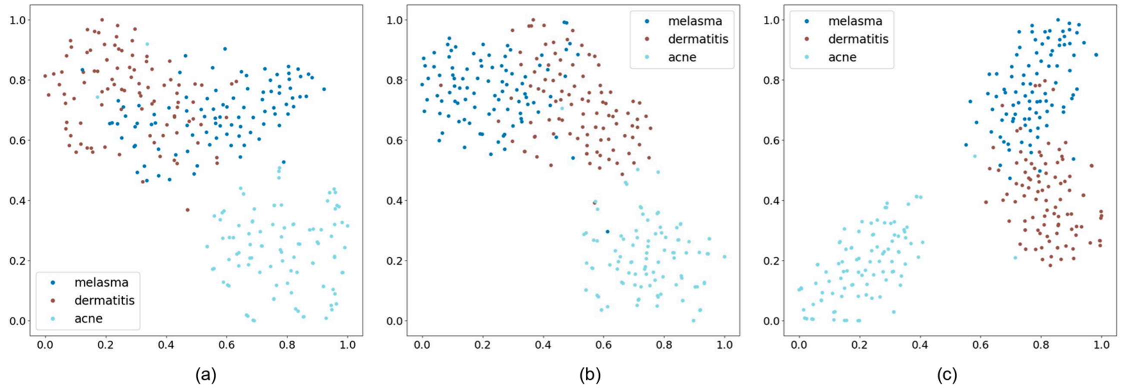

Figure S3. The t-SNE visualization of the DD dataset under different fusion methods. (a) The t-SNE visualization under early fusion. (b) The t-SNE visualization under late fusion. (c) The t-SNE visualization under the information-switching method.

Figure S4 displays the t-SNE visualization outcomes of the MM dataset using various fusion methods. The classification results of the early and late fusion models show that certain characteristics of MM samples are intermingled with the NMSC cluster. There is a notable degree of feature confusion between MM and NMSC, with our proposed model demonstrating superior distinguishing capabilities compared to the former two.

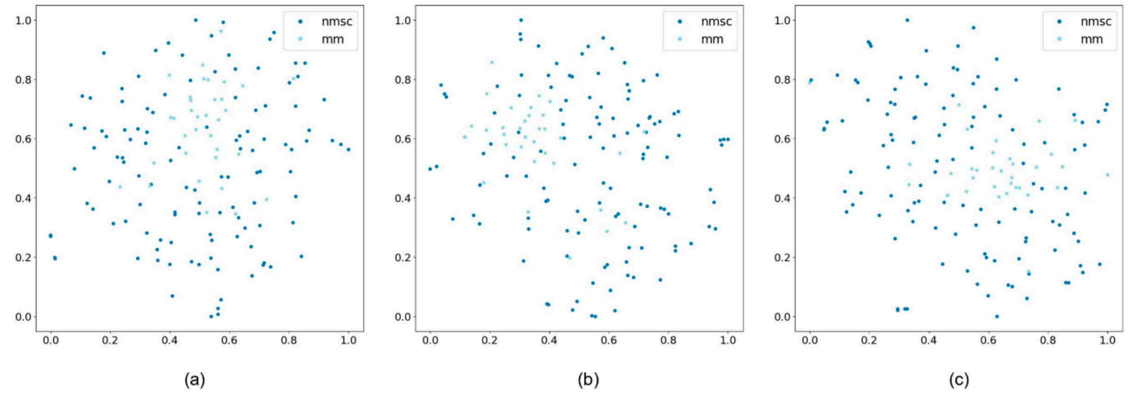

Figure S4. The t-SNE visualization of the MM dataset under different fusion methods. (a) The t-SNE visualization under early fusion. (b) The t-SNE visualization under late fusion. (c) The t-SNE visualization under the information-switching method.

Figure S5 displays the CM results of the DD dataset under various fusion methods. The proposed information-switching approach not only effectively distinguishes acne from the other two categories but also exhibits fewer misidentifications for dermatitis and melasma. This demonstrates that our proposed MDSIS-Net model performs best in the diagnostic differentiation task.

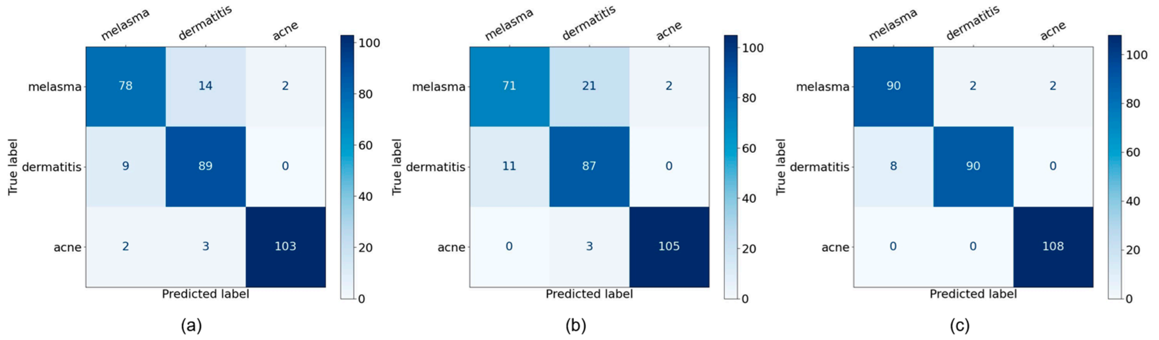

Figure S5. The CM visualization of the DD dataset under different fusion methods. (a) The CM visualization under early fusion. (b) The CM visualization under late fusion. (c) The CM visualization under the information-switching method.

Figure S6 presents the confusion matrix results of the DD dataset using different fusion methods. The information-switching approach proposed in this study shows better differentiation between MM and NMSC compared to the other methods, indicating that our MDSIS-Net model excels in the diagnostic differentiation task.

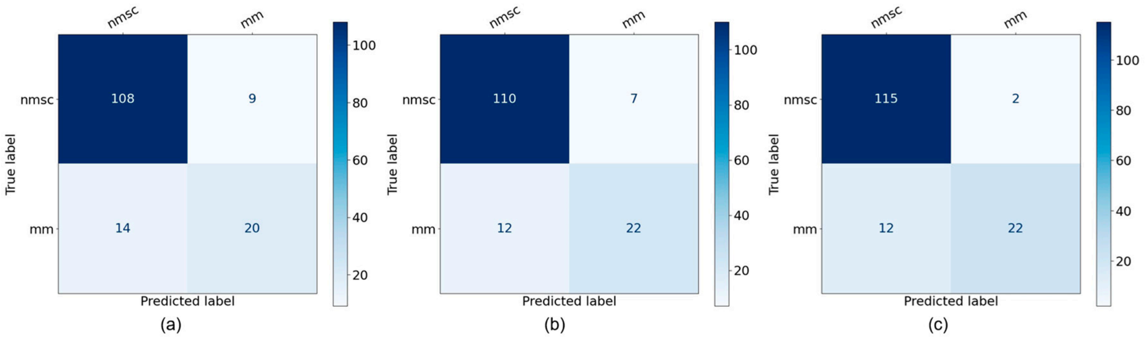

Figure S6. The CM visualization of the MM dataset under different fusion methods. (a) The CM visualization under early fusion. (b) The CM visualization under late fusion. (c) The CM visualization under the information-switching method.

Influence of modal fusion ratios on information switching

This study further conducts ablation experiments to investigate the impact of the exchange ratio between the current modality and other modalities. Table S2 presents the results for different exchange ratios. In the table, α represents the ratio of each information-switching branch's self-modality feature. The results show that the classification performance is the best when $\alpha=0.6$. This configuration outperforms the other two parameter settings by achieving a higher accuracy of 4.0% and 10.7%, a precision improvement of 3.6% and 9.1%, a recall enhancement of 3.7% and 7.7%, and an f1-score increase of 3.6% and 8.4% on the DD dataset. Additionally, mAP decreases by 0.6% compared to $\alpha=0.2$ and improves by 0.6% compared to $\alpha=0.8$. On the MM dataset, this configuration exhibits superior performance with an mAP increase of 2.5% and 1.1%, an accuracy increase of 2.6% and 3.9%, a precision boost of 5.9% and 9.4%, a recall rise of 2.8% and 2.6%, and an f1-score improvement of 3.8% and 4.9%. These findings indicate that appropriate tuning of parameter "a" can also lead to improved classification performance.

Table S2. Ablation analysis on several datasets using various modal fusion ratios.

α	mAP	Accuracy	Precision	Recall	F1-score	Dataset
0.2	**0.973**	0.920	0.899	0.923	0.911	DD
0.8	0.961	0.853	0.844	0.883	0.863	DD
0.6	0.967	**0.960**	**0.935**	**0.960**	**0.947**	DD
0.2	0.852	0.881	0.852	0.787	0.813	MM
0.8	0.866	0.868	0.817	0.789	0.802	MM
0.6	**0.877**	**0.907**	**0.911**	**0.815**	**0.851**	MM

Figure S7 illustrates the Grad-CAM visualizations of DD for different switching ratios in multi-modal information switching. When $\alpha=0.2$, the Grad-CAM highlights fail to focus on subtle inflammation, and non-facial areas are also significantly attended to. Conversely, with $\alpha=0.8$ and $\alpha=0.6$, the visualizations effectively capture the inflammatory regions within the red marks, as well as the discoloration areas of UV spots, porphyrins, and brown spots. Notably, the region of interest is more pronouncedly red with greater feature distinction at $\alpha=0.6$.

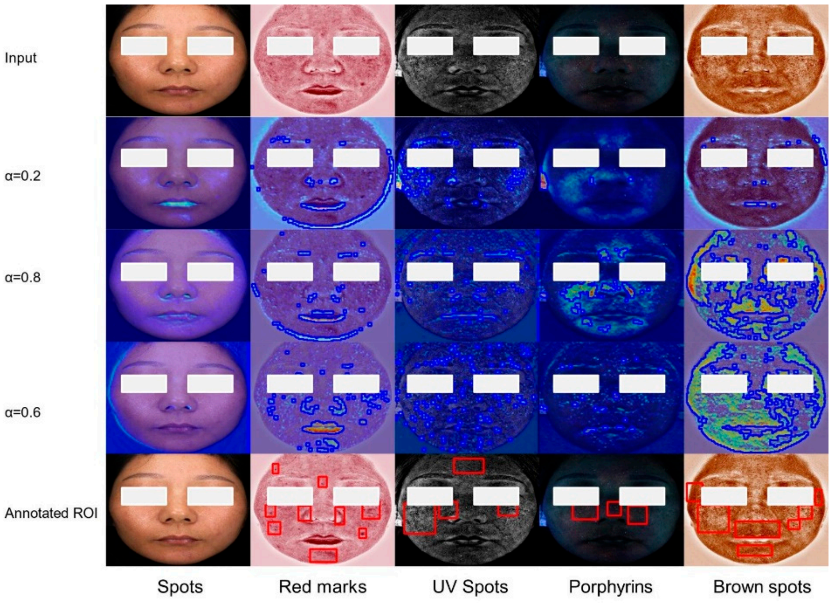

Figure S7. Comparison of the feature maps on different fusion ratios for the DD dataset.

Figure S8 illustrates the Grad-CAM visualizations of MM for different switching ratios in multi-modal information switching. Our proposed information-switching model effectively integrates information from both clinical and dermoscopic images at α=0.6 , accurately identifying regular pigmented regions and asymmetrical rash areas, as illustrated by the red regions in Figure S8.

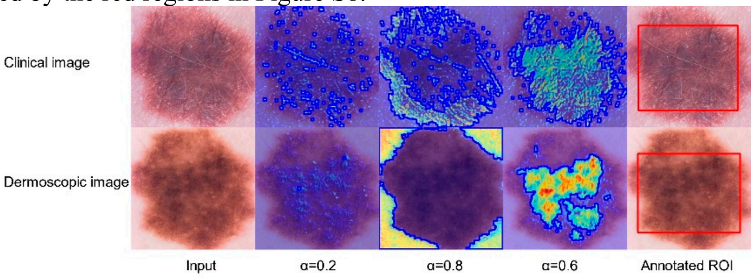

Figure S8. Comparison of the feature maps on different fusion ratios for the MM dataset.

Figure S9 displays the t-SNE visualization results of DD under different fusion ratios during multi-modal information switching. From the figure, it can be observed that, although the distances between acne and dermatitis as well as melasma are emphasized under the first two parameter settings, the distance between dermatitis and melasma is further increased, causing more confusion between these two classes. This indicates that under the parameter values of α=0.6 , the distances between multiple categories are more evenly balanced in terms of their features.

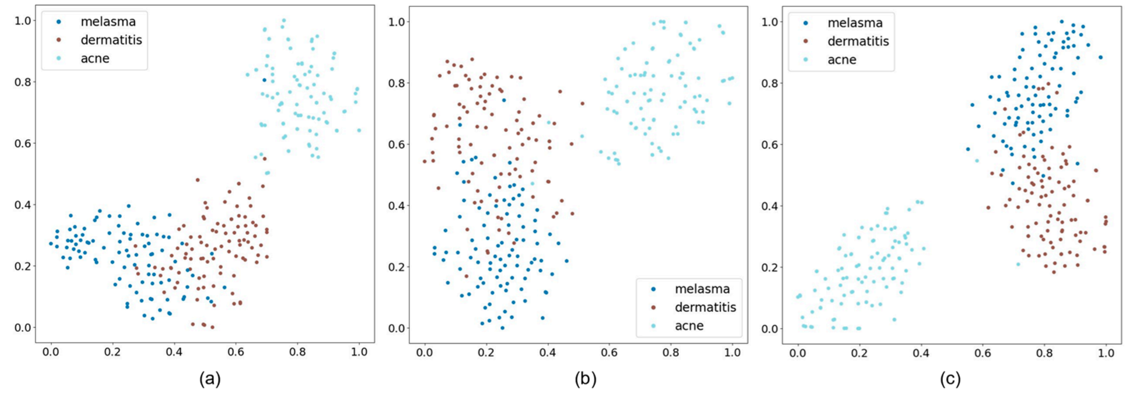

Figure S9. The t-SNE visualization of the DD dataset on different fusion ratios. (a) The t-SNE visualization at a=0.2. (b) The t-SNE visualization at a=0.8. (c) The t-SNE visualization at a=0.6.

Figure S10 displays the t-SNE visualization outcomes of the MM dataset under different fusion ratios. Fusion rations at $\alpha=0.2$ and $\alpha=0.8$ reveals that certain characteristics of MM samples are blended with the NMSC cluster. There is a significant level of feature overlap between MM and NMSC, with our proposed model exhibiting superior distinguishing abilities compared to the former two.

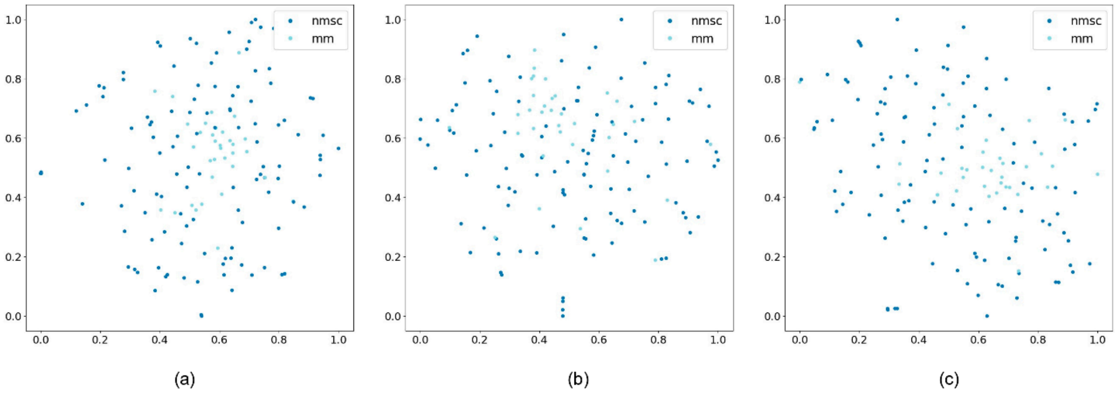

Figure S10. The t-SNE visualization of the MM dataset on different fusion ratios. (a) The t-SNE visualization at a=0.2. (b) The t-SNE visualization at a=0.8. (c) The t-SNE visualization at a=0.6.

Figure S11 displays the CM results of DD under different ratios during multi-modal information switching. When setting $\alpha=0.6$, the proposed MDSIS-Net model not only successfully differentiates acne from the other two categories but also shows fewer misclassifications between dermatitis and melasma. This confirms the superior performance of our MDSIS-Net model in discriminating diagnosis.

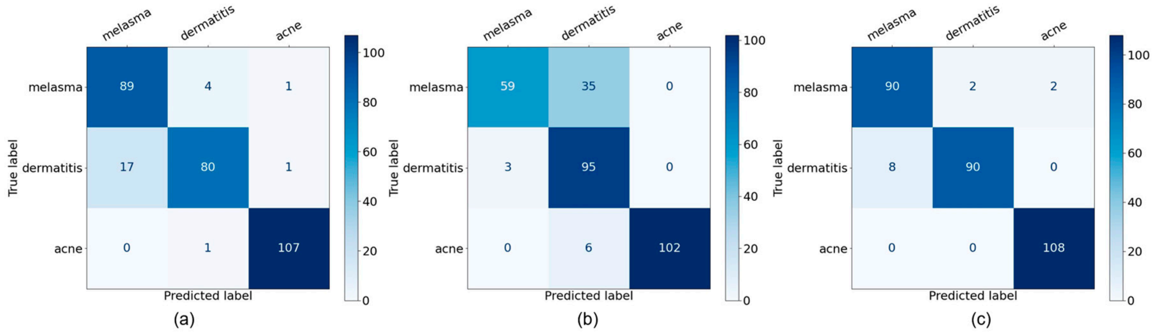

Figure S11. The CM visualization of the DD dataset on different fusion ratios. (a) The CM visualization at a=0.2. (b) The CM visualization at a=0.8. (c) The CM visualization at a=0.6.

Figure S12 presents the CM results of the MM dataset using different fusion ratios. The fusion ratio at α=0.6 in this study shows better differentiation between MM and NMSC compared to the other two.

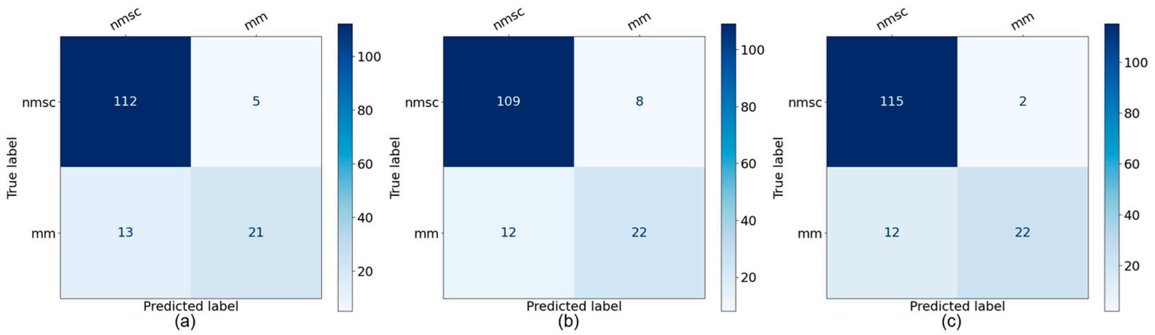

Figure S12. The CM visualization of the MM dataset on different fusion ratios. (a) The CM visualization at a=0.2. (b) The CM visualization at a=0.8. (c) The CM visualization at a=0.6.

The impact of the number of layers in information switching

This study further investigates the effect of different layers in multi-modal information switching, conducting ablation experiments by comparing four distinct methods. The first method involves information switching for the first four layers of the CNN, while the second method switches information for the last four layers. The third method switches information for the even-numbered layers, and the fourth method involves switching information for all layers of the CNN. Table S3 demonstrates the performance of each method in terms of mAP, accuracy, precision, recall, and f1-score. Results indicate that performing information switching on all layers of the CNN yields better performance compared to the other three methods, with an increase of 1.7%, 0.6%, and 0.3% in mAP, 2.7%, 3.7%, and 2.7% in accuracy, 1.6%, 4.8%, and 2.5% in precision, 2.0%, 1.7%, and 2.0% in recall, and 1.8%, 3.3%, and 2.4% in f1-score on the DD dataset, respectively. Performing on all layers of the CNN yields better performance compared to the other three methods, with an increase of 2.7%, 3.8%, and 1.3% in mAP, 1.3%, 1.3%, and 2.0% in accuracy, 2.5%, 1.1%, and 3.1% in precision, 1.9%, 2.9%, and 3.4% in recall, and 2.2%, 2.6%, and 3.5% in f1-score on the MM dataset, respectively. Information switching for all layers yields better results than the other three methods.

Table S3. Ablation experiments on the number of layers in information switching across different datasets.

Information switching layers	mAP	Accuracy	Precision	Recall	F1-score	Dataset
The first four layers	0.950	0.933	0.919	0.940	0.929	DD
The last four layers	0.961	0.923	0.887	0.943	0.914	DD
Layer 2, 4, and 6	0.964	0.933	0.910	0.940	0.925	DD
All layers	**0.967**	**0.960**	**0.935**	**0.960**	**0.947**	DD
The first four layers	0.850	0.894	0.886	0.796	0.829	MM
The last four layers	0.839	0.894	0.900	0.786	0.825	MM
Layer 2, 4, and 6	0.864	0.887	0.880	0.781	0.816	MM
All layers	**0.877**	**0.907**	**0.911**	**0.815**	**0.851**	MM

Figure S13 illustrates the Grad-CAM visualizations of DD for the number of layers of information switching. With all layers information switching, the visualizations effectively capture the inflammatory regions within the red marks, as well as the discoloration areas of UV spots, and brown spots. It's particularly noteworthy that the region of interest stands out more vividly in red, showcasing enhanced feature differentiation across all layers.

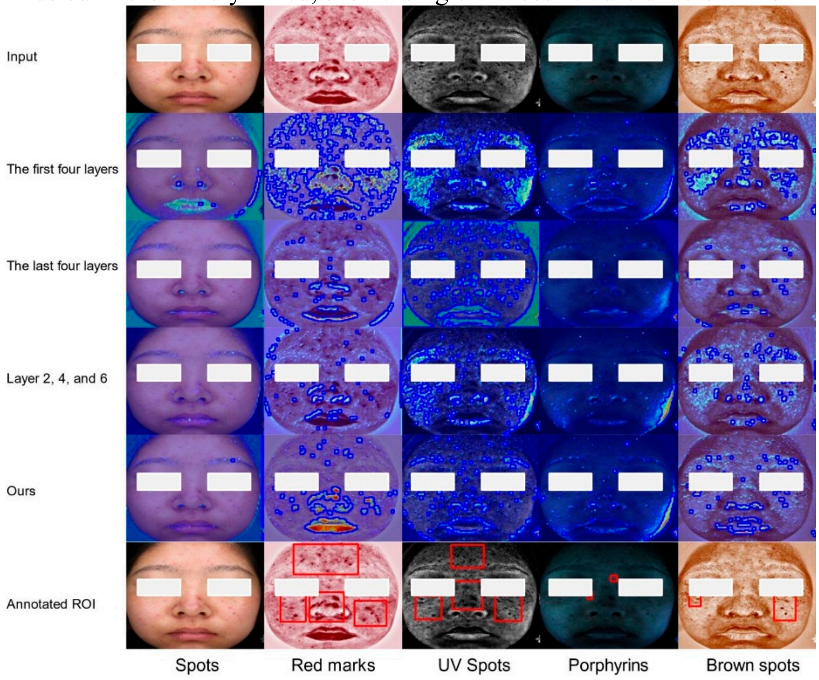

Figure S13. Effect of the number of information-switching layers for the DD dataset.

Figure S14 illustrates the Grad-CAM visualizations of MM for different switching ratios in multi-modal information switching. Our proposed model effectively integrates information from both clinical and dermoscopic images with all layers of information switching, accurately identifying regular pigmented regions and asymmetrical rash areas, as illustrated by the red regions in Figure S14.

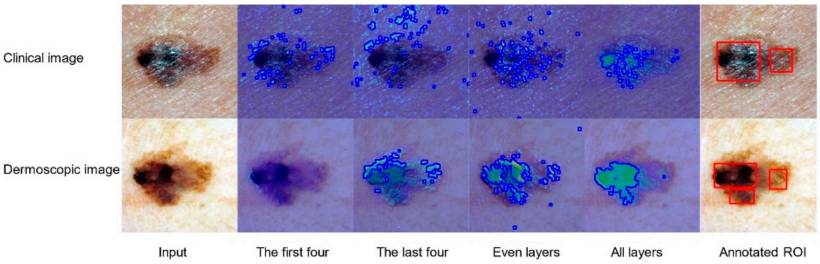

Figure S14. Effect of the number of information-switching layers for the MM dataset.

Figure S15 presents the t-SNE visualization results of DD under different layers of information switching in multi-modal data. From the graph, it is observed that performing information switching on all layers of the CNN leads to a wider separation between the 'acne' and 'dermatitis' as well as 'melasma' categories, with reduced confusion between the 'dermatitis' and 'melasma' categories. This indicates that performing information switching on all layers of the CNN achieves a more balanced distance between features of different categories. These findings highlight the importance of considering information from all layers for achieving optimal performance in the multi-modal information switching task.

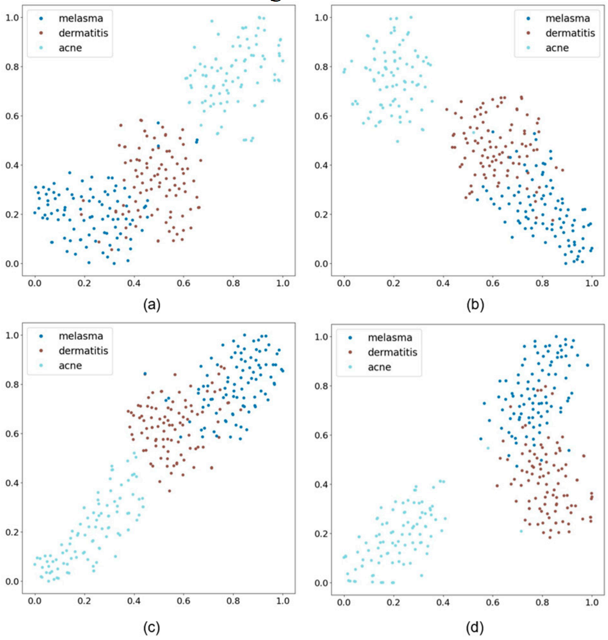

Figure S15. The t-SNE visualization of the DD dataset on different layers in multi-modal information switching. (a) The t-SNE visualization with the first four layers. (b) The t-SNE visualization with the last four layers. (c) The t-SNE visualization with even-numbered layers. (d) The t-SNE visualization with all layers.

Figure S16 displays the t-SNE visualization outcomes of the MM dataset under different fusion ratios. The former three information-switching layers reveal that certain characteristics of MM samples are blended with the NMSC cluster. There is a significant level of feature overlap between MM and NMSC. Our proposed model and using all layers for information exchange exhibit superior distinguishing abilities compared to the former three.

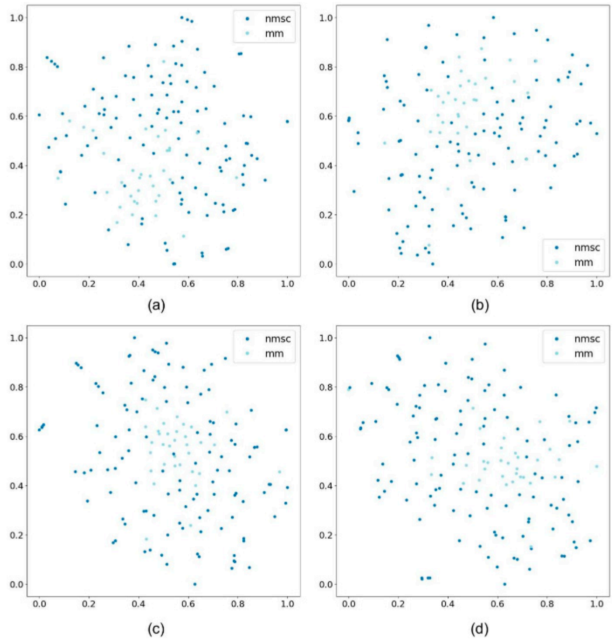

Figure S16. The t-SNE visualization of the MM dataset on different layers in multi-modal information switching. (a) The t-SNE visualization with the first four layers. (b) The t-SNE visualization with the last four layers. (c) The t-SNE visualization with even-numbered layers. (d) The t-SNE visualization with all layers.

Figure S17 displays CM results obtained under different layers of information switching in the multi-modal data. It is observed that when performing information switching on all layers of the CNN, not only does it successfully differentiate the 'acne' category from the other two categories, but it also leads to reduced misclassifications between the 'dermatitis' and 'melasma' categories. This highlights the effectiveness of utilizing information from all layers in the CNN for accurate classification in the multi-modal information switching task.

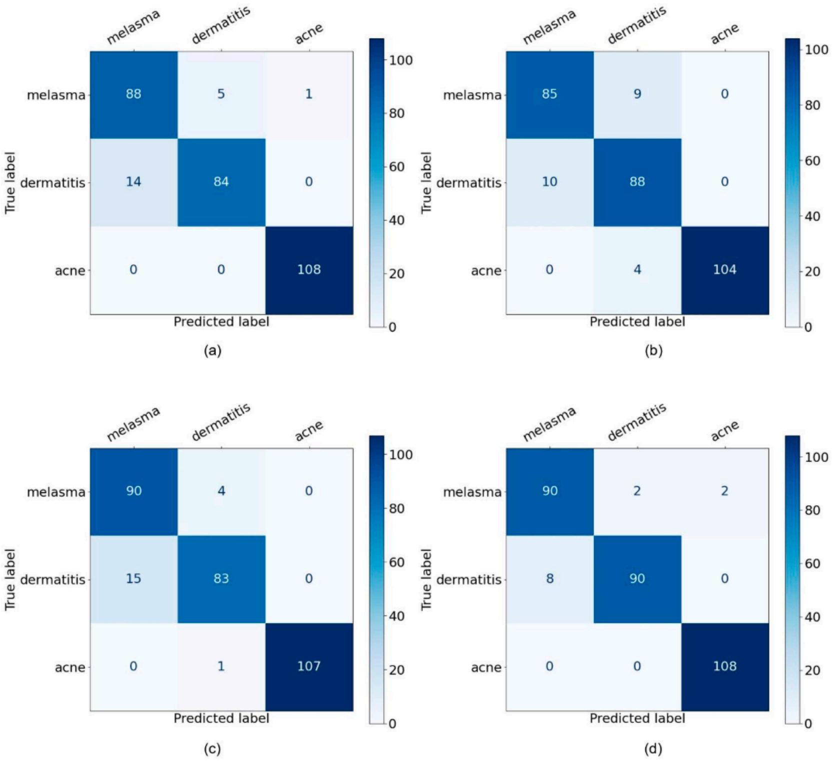

Figure S17. The CM visualization of the DD dataset on different layers in multi-modal information switching. (a) The CM visualization with the first four layers. (b) The CM visualization with the last four layers. (c) The CM visualization with even-numbered layers. (d) The CM visualization with all layers.

Figure S18 presents the confusion matrix results of the MM dataset under different layers of information switching in the multi-modal data. All layers for information switching in this study show better differentiation between MM and NMSC compared to the other three.

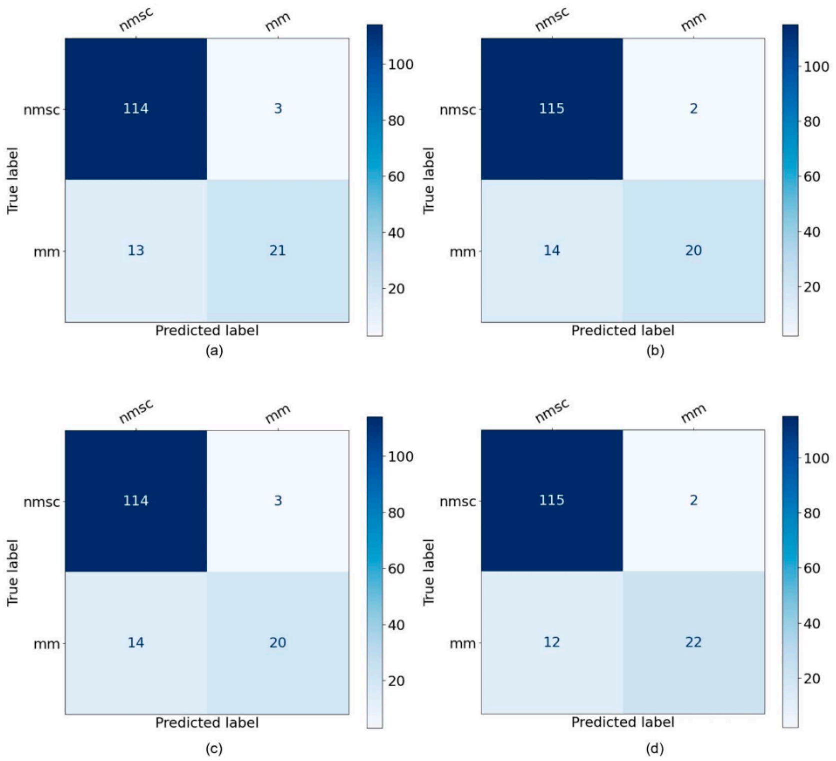

Figure S18. The CM visualization of the MM dataset on different layers in multi-modal information switching. (a) The CM visualization with the first four layers. (b) The CM visualization with the last four layers. (c) The CM visualization with even-numbered layers. (d) The CM visualization with all layers.

Model sensitivity to data variations

The model's performance is evaluated under different data variations, including changes in input size, blur, and the presence of noise. As shown in Table S4, the best performance is achieved with an input size of 384 × 384 pixels, yielding an accuracy of 0.960 on the DD dataset and 0.907 on the MM dataset. However, since image-based clinical diagnosis places high demands on image quality, performance slightly declines with average blur and Gaussian noise, indicating that while the model is robust to common data variations, it can be affected by poor image quality.

Table S4. Model performance under different data variations.

Data variation type	Input Size	Accuracy	Precision	Recall	Dataset
Original data	224	0.913	0.902	0.917	DD
Original data	304	0.937	0.919	0.950	DD
Original data	384	**0.960**	**0.935**	**0.960**	**DD**

Average blur	384	0.860	0.832	0.877	DD
Gaussian noise	384	0.873	0.867	0.910	DD
Original data	224	0.874	0.854	0.762	MM
Original data	304	0.894	0.875	0.806	MM
Original data	384	**0.907**	**0.911**	**0.815**	**MM**
Average blur	384	0.868	0.847	0.748	MM
Gaussian noise	384	0.887	0.868	0.792	MM

Performance with fewer modalities

The model's performance improves significantly with more modalities. As shown in Table S5, using all five modalities on the DD dataset results in the highest accuracy (0.960), while using only one modality (spots) reduces accuracy to 0.893. Similarly, on the MM dataset, using both modalities achieves an accuracy of 0.907, whereas using only the dermoscopic image drops accuracy to 0.868. These results highlight the importance of incorporating multiple modalities for better performance.

Table S5. Model performance under different numbers of modalities.

Number of modalities	Modality combination	Accuracy	Precision	Recall	Dataset
5	All modalities	**0.960**	**0.935**	**0.960**	DD
3	Spots, red masks, and UV spots	0.920	0.913	0.943	DD
1	Spots	0.893	0.878	0.933	DD
2	Both modalities	**0.907**	**0.911**	**0.815**	MM
1	Dermoscopic image	0.868	0.810	0.810	MM

Performance with shifting data distributions

The model's performance is tested under different data splits. As shown in Table S6, the best results are achieved with a 70%-15%-15% split, yielding an accuracy of 0.960 on the DD dataset and 0.907 on the MM dataset. Performance declines with smaller training sets, such as a 50%-25%-25% split, where accuracy drops to 0.916 on the DD dataset and 0.794 on the MM dataset. This highlights the sensitivity of the model to the distribution of data across training, validation, and testing sets, and demonstrates that increasing the amount of training data can enhance the model's performance.

Table S6. Model performance under different data splits.

Split ratio (Training/Validation/Testing)	Accuracy	Precision	Recall	Dataset
70%/15%/15%	**0.960**	**0.935**	**0.960**	DD
60%/20%/20%	0.925	0.915	0.935	DD
50%/25%/25%	0.916	0.903	0.924	DD
70%/15%/15%	**0.907**	**0.911**	**0.815**	MM
60%/20%/20%	0.812	0.810	0.678	MM

50%/25%/25%	0.794	0.750	0.691	MM

Performance across training, validation, and test sets

The model's performance is evaluated across training, validation, and test sets as shown in Table S7. On the DD dataset, it achieves accuracies of 0.995 (training), 0.964 (validation), and 0.960 (testing), with precision and recall consistently above 0.935. On the MM dataset, accuracies are 0.994 (training), 0.908 (validation), and 0.907 (testing), with high precision and recall values. These results demonstrate the model's robustness and consistent performance, validating its suitability. A slight performance decline from training to testing is expected.

Table S7. Performance comparison of the proposed model on DD and MM datasets across training, validation, and test sets.

Phase	Accuracy	Precision	Recall	Dataset
Training	**0.995**	**0.992**	**0.999**	DD
Validation	0.964	0.945	0.970	DD
Test	0.960	0.935	0.960	DD
Training	**0.994**	**0.994**	**0.991**	MM
Validation	0.908	0.899	0.826	MM
Test	0.907	0.911	0.815	MM

Model Architecture

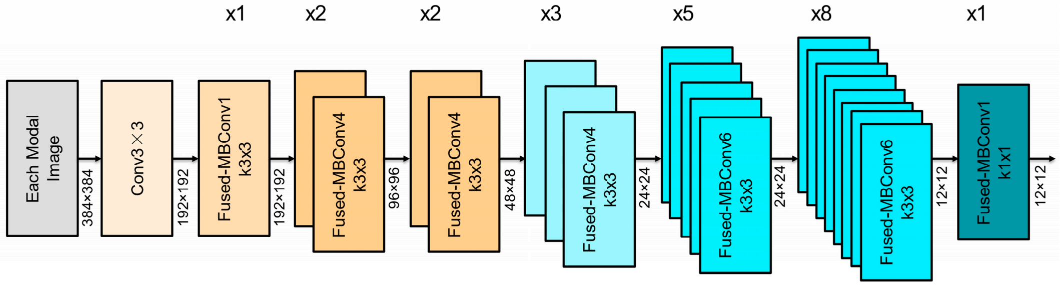

Figure S19. An overview of the structure used for intra-modality feature extraction.

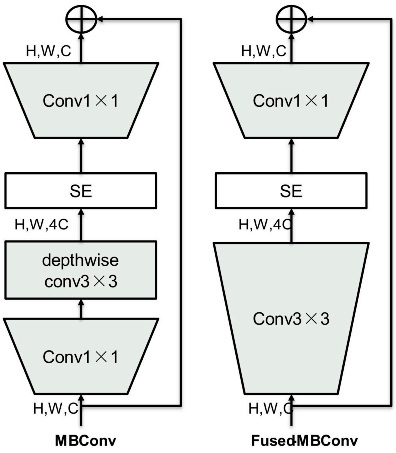

Figure S20. The MBConv and Fused-MBConv module's detailed construction.

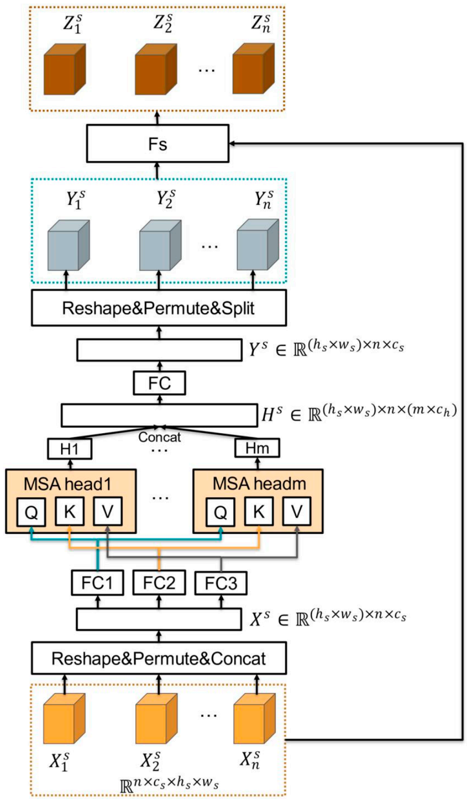

Figure S21. Detailed MDSIS-Net architecture illustrating a particular multi-modal information switching network implementation.

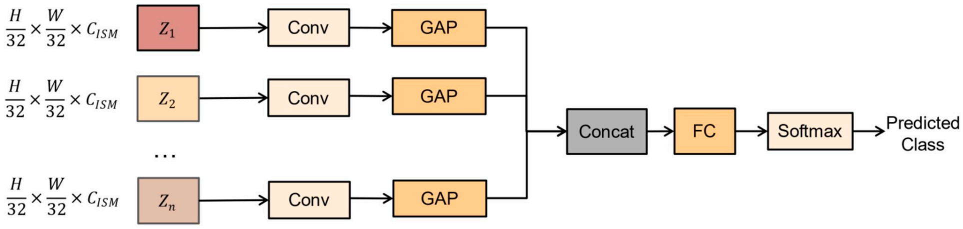

Figure S22. The general structure for the feature aggregation and classification.

Performance analysis of our MDSIS-Net

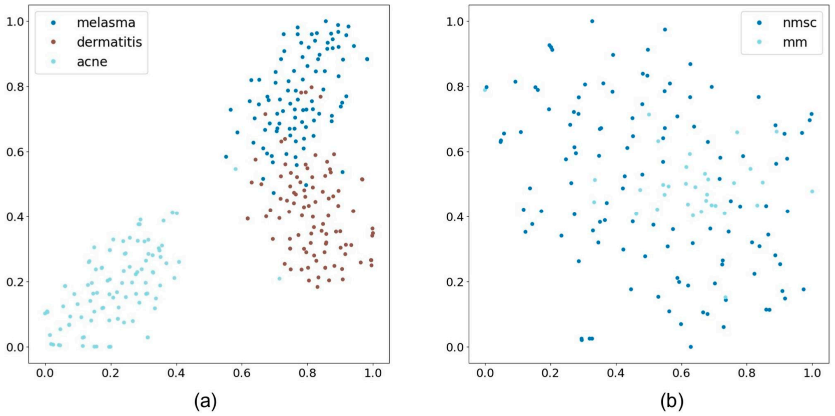

Figure S23. The t-SNE visualization of DD and MM datasets. (a) The t-SNE result for DD. (b) The t-SNE result for MM.

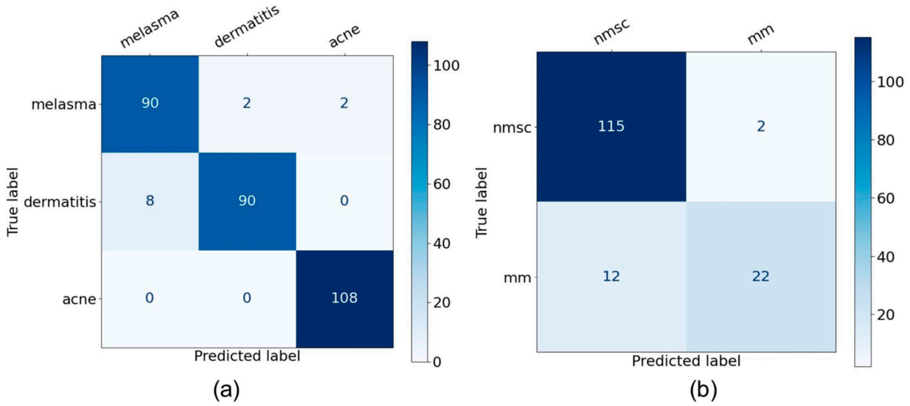

Figure S24. The CM visualization of DD and MM datasets. (a) The CM result for DD. (b) The CM result for MM.
